# Detecting and characterizing creeping fat in Crohn’s disease: agreement between intestinal ultrasound and computed tomography enterography

**DOI:** 10.1186/s13244-024-01807-4

**Published:** 2024-09-23

**Authors:** Mengyuan Zhou, Zihan Niu, Li Ma, Wenbo Li, Mengsu Xiao, Yudi He, Jing Qin, Yuxin Jiang, Wei Liu, Qingli Zhu

**Affiliations:** 1 Department of Ultrasound, Peking Union Medical College Hospital, Chinese Academy of Medical Sciences and Peking Union Medical College, 100730 Beijing, China; 2 Department of Radiology, Peking Union Medical College Hospital, Chinese Academy of Medical Sciences and Peking Union Medical College, 100730 Beijing, China

**Keywords:** Creeping fat, Crohn’s disease, Ultrasound, Computed tomography enterography

## Abstract

**Objectives:**

Creeping fat (CF) is associated with stricture formation in Crohn’s disease (CD). This study evaluated the feasibility of intestinal ultrasound (IUS) for semiquantitative analysis of CF and compared the agreement between IUS and computed tomography enterography (CTE).

**Methods:**

In this retrospective study, we recruited consecutive CD patients who underwent IUS and CTE. CF wrapping angle was analyzed on the most affected bowel segment and was independently evaluated by IUS and CTE. We evaluated the wrapping angle of CF in the cross- and vertical sections of the diseased bowel. CF wrapping angle was divided into < 180° and ≥ 180°. IUS performance was assessed using CTE as a reference standard, and IUS interobserver consistency was evaluated.

**Results:**

We enrolled 96 patients. CTE showed that CF wrapping angle was < 180° in 35 patients and ≥ 180° in 61 patients. We excluded three cases in which the observation positions were inconsistent between the IUS and CTE. Excellent agreement was shown between US and CTE (82/93, 88.2%). The eleven remaining cases showed inconsistencies mostly in the terminal ileum (*n* = 5) and small intestine (*n* = 4). Total agreement between IUS observers was 89.6% (86/96, κ = 0.839, *p* = 0.000), with perfect agreement for the ileocecal and colonic segments (35/37, 94.6% and 20/21, 95.2%, respectively) and moderate agreement for small intestinal segments (16/21, 76.2%).

**Conclusions:**

IUS could be of value and complementary to CTE for assessing CF, particularly in patients with affected terminal ileum and colon. IUS is a non-invasive technique for monitoring CD patients.

**Critical relevance statement:**

In our study, excellent agreement was shown between intestinal US observers as well as between US and CT enterography (CTE) for assessing creeping fat (CF), which showed that ultrasound could be of value and complementary to CTE.

**Key Points:**

- Creeping fat (CF) is a potential therapeutic target in Crohn’s disease.
- Excellent agreement was shown between US and CT Enterography (CTE) for assessing CF.
- Ultrasound could be complementary to CTE for assessing CF.

**Graphical Abstract:**

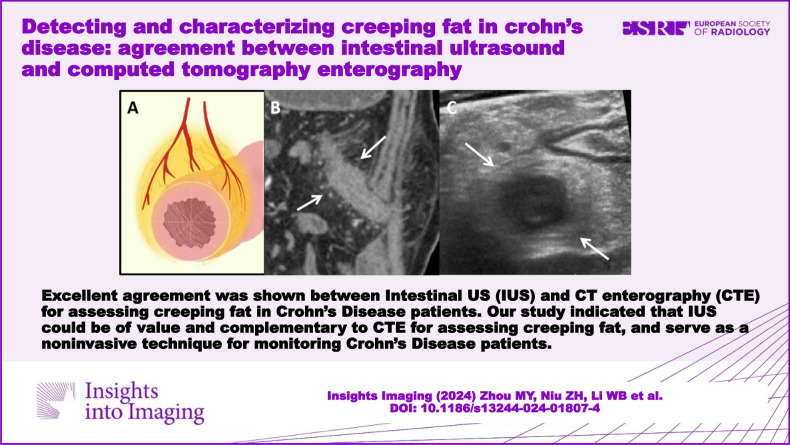

## Introduction

Intestinal stenosis is an important complication of Crohn’s disease (CD) and seriously affects the quality of life and prognosis of patients. Mesenteric fat wrapping around the inflamed gut, known as creeping fat (CF), is a hallmark of CD. As a potent producer of fatty acids, cytokines, growth factors and adipokines, CF plays an important role in the regulation of immunity and inflammation in CD, where surgical histopathological evaluation shows a significant association between CF and connective tissue changes in the bowel wall, such as muscular hypertrophy, fibrosis, and stricture formation [1]. Increasing evidence suggests that CF is associated with stricture formation in CD, making it a potential therapeutic target [2]. Therefore, non-invasive imaging approaches for detecting CF in patients with CD have attracted attention in recent years.

Computed tomography enterography (CTE) is a potentially non-invasive tool for evaluating CF in CD. Li et al [3] developed and characterized a novel mesenteric creeping fat index (MCFI) based on CT in CD patients. As a semiquantitative measure, MCFI was graded based on the extent to which mesenteric fat extended around the intestinal circumference, using the vessels in the fat as a marker. An excellent correlation was shown between MCFI and the extent of fat wrapping in specimens (*r* = 0.840, *p* = 0.000). Zhou et al [4] confirmed that MCFI was associated with early postoperative recurrence. Among all variables, MCFI contributes the optimal AUC (0.838 (0.758–0.919)) and is an independent predictor of early postoperative recurrence (OR = 25.71 (7.65–86.35), *p* < 0.001).

Intestinal ultrasonography (IUS) is a non-invasive, highly accurate imaging method. The occurrence of mesenteric fat proliferation on IUS is considered to be a key imaging feature of intestinal inflammatory activity in CD [5–7]. In the study of Bhatnagar et al [8], twelve CD patients underwent small bowel US prior to resection, and the association between sonographic observations and histopathological scores was examined in 50 selected bowel cross-sections. The mesenteric fat echogenicity was significantly associated with acute and chronic inflammation. However, the quantitative definition of CF by US and its comparison with other imaging methods have not yet been reported.

This study was a semiquantitative analysis of CF using IUS in patients with CD and a comparison with the findings with CTE.

## Materials and methods

### Study population

This was a single-center retrospective study that included data on consecutive patients with CD from June 2022 to June 2023 at the Department of Gastroenterology, Peking Union Medical College Hospital, China. The inclusion criteria were: (1) patients with a confirmed diagnosis of CD based on comprehensive clinical, endoscopic, imaging and histological criteria [2, 9]; (2) patients hospitalized due to newly developed intestinal inflammatory activity; and (3) patients who underwent IUS and CTE examinations at an interval of < 2 weeks. According to the ECCO-ESGAR guidelines [10], all newly diagnosed CD patients should undergo small bowel assessment (intestinal ultrasound, MR enterography and/or capsule endoscopy). Cross-sectional imaging can detect internal penetrating disease and intra-abdominal abscesses with varying accuracy, and small bowel involvement in CD patients. MRI/CTE is preferable to ultrasound for deep-seated fistulae or abscesses. Therefore, MRE or CTE combined with IUS examination was performed for comprehensive evaluation in CD patients with the newly flared disease. Referred to MCFI, we included patients who underwent both CTE and US examinations.

**Fig. 1:**
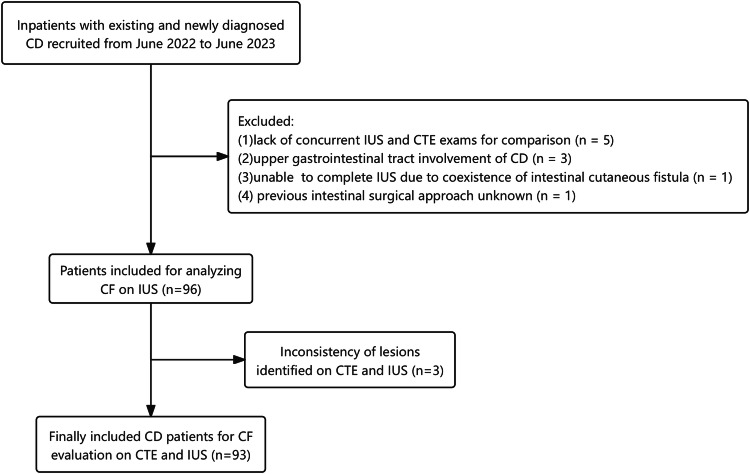
Study flowchart. CD, Crohn’s disease; CTE, computed tomography enterography; IUS, intestinal ultrasound

The exclusion criteria were: (1) upper gastrointestinal tract involvement of CD; (2) coexistence of pathologies such as intestinal adhesion/fistula/abscess, which affected the assessment of US and CTE; (3) patients with a history of prior enterectomy with unknown specific surgical methods; and (4) patients with incomplete clinical and imaging data. This study was approved by the Medical Ethics Committee of Peking Union Medical College Hospital, and the requirement for informed consent was waived. Figure 1 shows the flowchart of patient enrollment.

The following baseline clinical characteristics were recorded: sex, age, course of disease, body mass index, smoking history, disease location and behavior, perianal disease, C-reactive protein and erythrocyte sedimentation rate. Montreal classification was accepted to classify the disease behavior of CD [11]. The Montreal classification of CD considered the age of onset (A), disease location (L), disease behavior (B), and perianal disease (p) as the predominant phenotypic elements. The B1 type is non-stricturing and non-penetrating, the B2 type is stricturing, and the B3 type is penetrating.

### CTE examination

All patients were required to fast for 6–8 h prior to CTE without the use of intestinal paralytic agent, followed by ingestion of 1.5–2.0 L 2.5% mannitol solution 45–60 min before image acquisition. All patients underwent contrast-enhanced CT (Aquilion One, pixel spacing 0.644/0.644 mm, slice thickness 1.0 mm; Canon Medical Systems) from the dome of the liver to the symphysis pubis during a breath-hold with the patient in the supine position. After plain scanning, 2 mL/kg nonionic contrast agent (Ultravist 370; Schering, Berlin, Germany) was injected intravenously at a rate of 3.5 mL/s using a dual-head power injector. Forty milliliters of 0.9% saline were administered immediately after injection of contrast agent at the same rate. The enteric phase scan was started 40–45 s after injection, and the venous phase scan was initiated 70 s after injection. CTE images were independently analyzed by a radiologist with 15 years of experience in bowel CT (W.L.), who was blinded to the clinical information and IUS results.

**Fig. 2:**
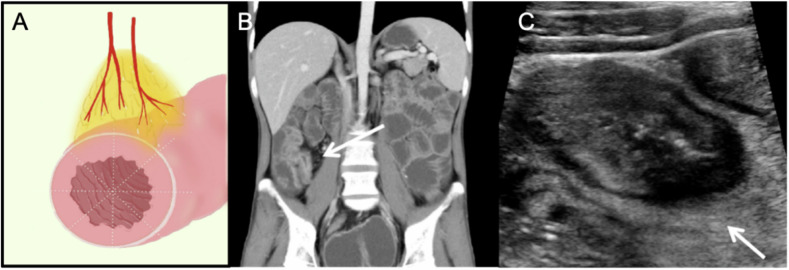
Schematic, CTE and IUS images from an 18-year-old male patient with Crohn’s disease. **A** The schematic diagram of transverse section of the intestine. The wrapping angle of creeping fat was < 180°. **B** In the coronal section of CTE (the 6th group of small intestine), there were straight small vessels on the mesenteric side (arrow), which indicated the wrapping angle of CF was < 180°. **C** The corresponding IUS image of the transverse section of the intestine, showing the coverage of the hyperechoic CF, was < 50% of the intestinal circumference. CF, creeping fat; CTE, computed tomography enterography; IUS, intestinal ultrasound

**Fig. 3:**
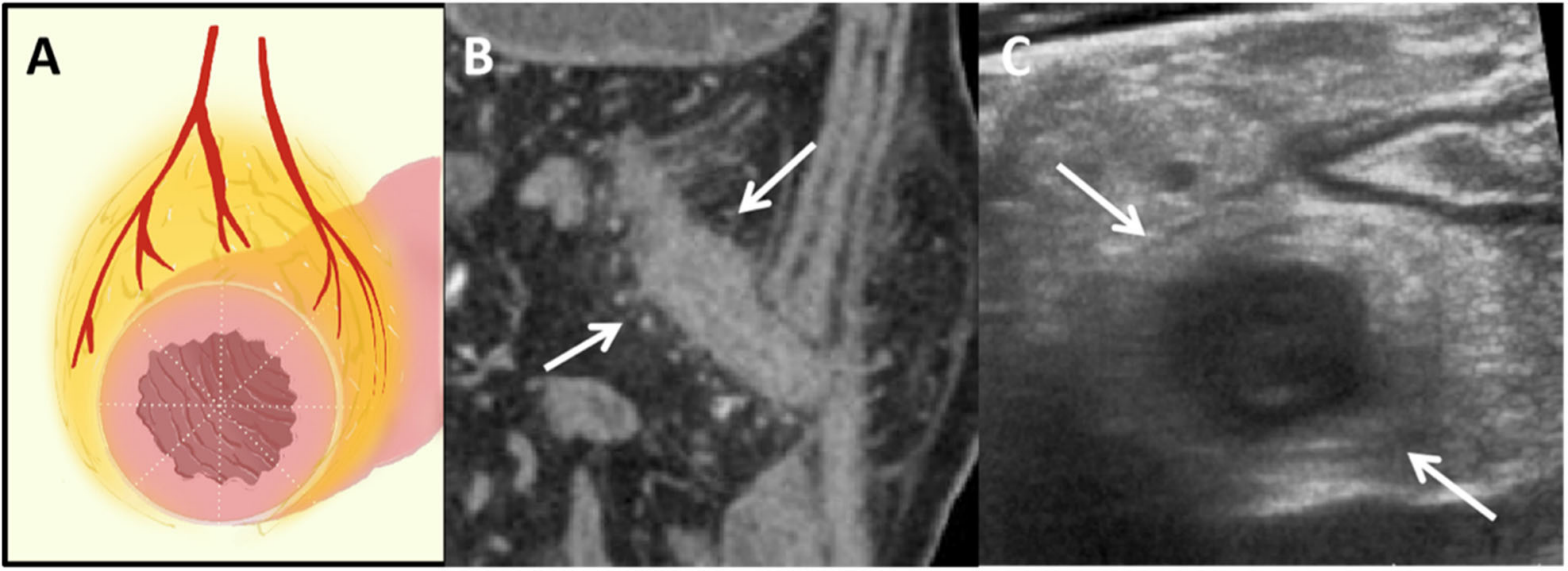
Schematic, CTE and IUS images from a 24-year-old male patient with Crohn’s disease. **A** Schematic diagram of transverse section of the intestine. The wrapping angle of CF was > 180°. **B** In the coronal section of CTE (descending colon), there were obvious straight small vessels on both the mesenteric side and opposite side of the mesentery (arrows), which indicated the wrapping angle of CF was > 180°. **C** IUS image of the transverse section of the intestine, which showed the hyperechoic CF surrounded the entire intestine (arrows). The patient underwent surgery 1 month later due to failed conservative management and worsened obstructive symptoms. CF, creeping fat; CTE, computed tomography enterography; IUS, intestinal ultrasound

In each patient, the bowel segment with the most luminal narrowing and/or the most wall thickening on CTE images was selected. The technical aspects of the MCFI, which can show CF, have been previously described in detail [3]. In our study, the extent of CF entanglement was characterized by the distribution of small straight mesenteric vessels around a diseased intestine. The severity of CF was graded based on the extent to which mesenteric fat extended around the intestinal circumference using the vessels in the fat as a marker, including the angle of CF wrapping (< 180° or ≥ 180°) (Figs. 2B and 3B). We recorded the observed segments and evaluated the wrapping angle of CF in the cross- and vertical sections of the diseased bowel. When fat wrapped and covered < 50% of the intestinal circumference, CF wrapping angle was < 180°, and when fat wrapped and covered ≥ 50% of the intestinal circumference of the intestine, the CF wrapping angle was ≥ 180°.

### IUS examination

Patients were examined after an overnight fast without the use of intestinal paralytic agent. IUS was performed by one of the three experienced radiologists (L.M., W.B.L., and M.S.X.), who were blinded to the CTE results, using a Philips type IU22 diagnostic system (Royal Philips Electronics Group, Netherlands) with low- (C5-1) and high (L9-3) frequency probes, and SuperSonic Imagine type Aixplorer diagnostic system (Aix Provence, France) with low- (XC6-1) and high- (SL10-2) frequency probes for overview and detailed examinations of the bowel wall, respectively. Both B-mode US and Color Doppler flow imaging were used in the evaluation of CF. We evaluated CF in the most severely diseased intestinal segment and recorded the location of the observed segments. The IUS images were independently analyzed by two experienced US physicians (M.Y.Z. and Z.H.N.), who were blinded to the CTE results. In the event of discordant diagnoses, a third expert was consulted (Q.L.Z.) until consensus was reached.

We adopted a region-to-region positioning approach between CTE and US. Location matching was performed by a radiologist with 15 years of imaging experience (W.L.) and three experienced US physicians (M.Y.Z., Z.H.N., and Q.L.Z.) in gastrointestinal disease diagnosis. All bowel segments were evaluated based on lesion characteristics (e.g., intestinal stenosis, fistulas, and abscesses), the thickest intestinal wall and the most abundant Doppler blood flow.

CF was defined as the hyperechoic structure surrounding the diseased bowel segment (Figs. 2A and 3A). The wrapping angle of CF was evaluated on the cross-section of the diseased bowel. As for CTE, we divided the patients into two groups according to the CF wrapping angle around the evaluated segment (< 180° or ≥ 180°) (Figs. 2C and 3C).

### Statistical analysis

We used SPSS version 26.0 (IBM Corporation, Armonk, NY, USA) for statistical analysis. Significance was set at two-sided *p* < 0.05. Normally distributed data were expressed as mean ± standard deviation, and non-normally distributed data were presented as median (interquartile range). Interobserver analyses were performed between IUS readers by calculating Fleiss κ values, which were interpreted as poor (κ < 0.2), fair (κ 0.2–0.4), moderate (κ 0.4–0.6), good (κ 0.6–0.8) and excellent (κ > 0.8) [12]. Agreement between IUS and CTE-based reference standards was assessed by calculating total agreement. Fisher’s precision probability test was used to compare whether there were significant differences around the groups with corresponding 95% CIs.

## Results

A total of 106 CD patients were initially involved. Ultimately, we included 96 patients clinically diagnosed with CD. Ten patients were excluded because they did not undergo CTE and US (*n* = 5), upper gastrointestinal tract involvement of Crohn’s disease (*n* = 3), the coexistence of intestinal cutaneous fistula (*n* = 1), and the unknown surgical method of prior intestinal surgery (*n* = 1).

### Clinical data

The median age of the patients was 34.0 years (range 16–72 years), including five preadolescents (5.2%) and 91 adults (94.8%). Seventy-five patients (78.1%) were male, and 21 (21.8%) were female. Thirty-four patients (34/96, 35.4%) had a history of smoking. Thirty-six patients (31/96, 32.2%) had perianal involvement. Further patient characteristics are presented in Table 1.

**Table 1:** Demographic and clinical characteristics in patients with Crohn’s disease

	Number
Sex, *n* (male/female)	75/21
Age, years [median (IQR)]	34.0 [16–72]
Disease duration, months [median (IQR)]	79 [1–312]
Smoking history, *n* (%)	34/96 (35.4)
BMI [median (IQR)]	19.74 [13.28–28.23]
Disease location, *n* (%)	
Ileal (L1)	20/96 (20.8)
Colonic (L2)	15/96 (15.6)
Ileocolonic (L3)	52/96 (54.1)
Ileal and upper disease (L1 + 4)	3/96 (3.1)
Ileocolonic and upper disease (L3 + 4)	6/96 (6.2)
Disease behavior, *n* (%)	
Non-stricturing, non-penetrating (B1)	15/96 (15.6)
Stricturing (B2)	43/96 (44.7)
Penetrating (B3)	10/96 (10.4)
Stricturing and penetrating (B2 + 3)	28/96 (29.1)
Perianal involvement	31/96 (32.2)
Previous surgery, *n*	22 (23.0)
Evaluated segment, *n* (%)	
Small intestine	21/93* (22.5)
Terminal ileum and ileocecum	37/93 (39.7)
Colon	19/93 (20.4)
Anastomosis	16/93 (17.2)
CRP, mg/L [median (IQR)]	10.24 [0.08–172.00]
ESR, mm/h [median (IQR)]	23 [2–75]

Numbers in parentheses are percentages. Numbers in square brackets represent ranges

*BMI* body mass index, *CRP* C-reactive protein, *ESR* erythrocyte sedimentation rate, *IQR* interquartile range

* There were three cases with inconsistencies between IUS and CTE. To ensure the reliability of CF evaluation, we excluded these cases from the original 96

### Agreement between IUS and CTE

CTE showed that the CF wrapping angle was < 180° in 35 patients, and ≥ 180° in 61 patients. We evaluated the IUS and CTE images independently and found that the observation positions of three patients were inconsistent. To ensure the reliability of CF evaluation, we excluded these cases. Among 93 patients, the terminal ileum and ileocecum were the most affected (most thickened) parts of the bowel (37/93, 39.7%) (Table 1). The overall observed agreement between IUS and CTE was 88.2% (82/93). In patients with CF wrapping angle < 180° on CTE, IUS showed that the wrapping angle of CF was < 180° in 25 of 32 patients (78.1%). Seven patients had inconsistent IUS and CTE results, including in the small intestine (*n* = 2), terminal ileum (*n* = 3), colon (*n* = 1) and anastomosis (*n* = 1). In these inconsistent cases, 85.7% manifested as stricture rather than penetration. In patients with CF wrapping angle ≥ 180° on CTE, 57 of 61 patients (93.4%) had consistent IUS and CTE results. The remaining four patients had inconsistencies in the small intestine (*n* = 2) and terminal ileum (*n* = 2). There was one case of nontransmural lesions in both the small intestine and terminal ileum. The agreement between IUS and CTE for different disease behaviors is presented in Table 2.

**Table 2:** Agreement between intestinal ultrasound and computed tomography enterography of different disease behaviors (*n* = 93)

Disease behavior	CTE			US		Consistence, *n*	Inconsistence, *n*	Agreement, %	*p*-value	95% CI
	CF < 180°	CF ≥ 180°		CF < 180°	CF ≥ 180°					
B1	5/14		9/14	5/14	9/14	12	2	85.7	0.023	1.561, 656.054
B2	18/42		24/42	14/42	28/42	36	6	85.7	0.000	6.289, 568.599
B3	4/10		6/10	5/10	5/10	9	1	90.0	0.048	1.003, 35.908
B2 + 3	5/27		22/27	5/27	22/27	25	2	92.6	0.001	4.306, 1638.785

### Interobserver agreement for IUS of CF

IUS showed that the CF wrapping angle was < 180° in 29 patients (30.2%) and ≥ 180° in 67 patients (69.7%). We compared the agreement between three different observers. Excellent agreement was achieved among different CF wrapping angles among the IUS and CTE (κ = 0.839, *p* = 0.000). Ten cases had inconsistent results, including in the small intestine (*n* = 5), anastomosis (*n* = 2), ileocecum (*n* = 2), and splenic flexure of the colon (*n* = 1). Total agreement between IUS observers was 89.6% (86/96), which was particularly high for the ileocecal and colonic segments (35/37, 94.6% and 20/21, 95.2%, respectively), good for anastomosis (15/17, 88.2%) and moderate for small intestinal segments (16/21, 76.2%). The interobserver agreement for IUS of different disease behaviors is presented in Table 3. We further calculated the proportion of CF wrapping angles in different disease behaviors of CD. It showed that the segment with intestinal stricture (disease behavior was B2 classification) had a higher proportion of CF wrapping angle ≥ 180° than that in the other two types (Table 4).

**Table 3:** Agreement between intestinal ultrasound observers of different disease behaviors (*n* = 96)

Disease behavior	Consistence, *n*	Inconsistence, *n*	Agreement, %	*p*-value	95% CI
B1	14	1	93.3	0.002	1.558, 64.198
B2	38	5	88.4	0.000	8.216, 803.544
B3	9	1	90	0.048	0.035, 1.154
B2 + 3	25	3	89.3	0.003	3.034, 581.434

**Table 4:** Differences in CF wrapping angle on IUS among different disease behavior subtypes (*n* = 96)

Disease behavior	CF < 180° (*n*, %)	CF ≥ 180° (*n*, %)	*p*-value
B1	5/15, 33.3	10/15, 66.7	0.239
B2	14/43, 32.6	29/43, 67.4	
B3	5/10, 50.0	5/10, 50.0	
B2 + 3	5/28, 17.9	23/28, 82.1	

## Discussion

Different imaging features (such as intestinal wall thickness, structure of intestinal wall, intestinal fistula, etc.) are of great value in evaluating the disease activity of CD. These features have also been reported in previous studies and have been confirmed to have good diagnostic accuracy and consistency on CT and US [13, 14]. However, the consistency between CTE and US on CF is not yet clear. So, we focused on CF and proposed our study, and the results also indicated that IUS is a valuable and practical method for evaluating CF. In the previous study [3], multiplanar reconstruction and maximum-intensity projection were used to show CF. MCFI is considered a quantitative imaging method to observe the morphology and extent of CF entanglement. MCFI divided the bowel circumference into eight equal areas for the target bowel segments on the reconstructed enhanced CTE images. The complexity limits its clinical application to some extent. Referred to MCFI, we defined a clear semiquantitative IUS indicator for CF. The evaluation combined the ultrasonic features of transverse and longitudinal sections. And it turned out to be highly consistent with CTE. Thus, IUS provided an accurate and feasible method for the semiquantitative analysis method for CF evaluation. The characteristics of no radiation and no need to use contrast agents significantly increase patient tolerance and safety. It is expected to play an important role in prognosis evaluation and predictive value for treatment.

US clearly showed the CF around the inflamed intestinal segments—a hyperechoic structure closely surrounding the diseased bowel. It is an extension of mesenteric fat, beginning at the intestinal hilum (i.e., the zone where the mesentery and the intestine intersect) and progressively surrounding the intestinal wall [1]. As the lesion progresses, the extent of coverage of the bowel circumference gradually increases. In our study, we adopted a combination of transverse and longitudinal sections to observe CF more comprehensively. After we clearly defined the CF on IUS and standardized the method of observation, we obtained excellent interobserver agreement, which indicated that it was feasible to use IUS to examine CF and follow it up. Additionally, the overall observed agreement of CF on IUS and CTE was good (88.2%), which implied that IUS could be a reliable non-irradiating imaging approach for detecting CF in patients with CD, especially for patients with wider CF coverage (agreement of 93.4%). IUS was also a practical and low-cost tool for evaluating patients with ileal or ileocolonic disease, particularly in active CD.

For CD patients with different disease behaviors, both IUS and CTE had good agreement, indicating that IUS was a widely applicable, complementary imaging method for evaluating CF. It is worth mentioning that the assessment by IUS and CTE of penetrating lesions was more accurate than that of non-penetrating lesions (91.9% vs 85.7%). Among the eleven cases with inconsistent results between IUS and CTE, eight (72.7%) presented as nontransmural disease behavior. The type of disease behavior affected the extension of CF to the peri-intestinal area, which may explain the inconsistent results. The agreement between IUS observers varied with lesion site; the agreement was lowest (16/21, 76.2%) for small intestinal lesions and highest for ileocecal and colonic lesions (35/37, 94.6% and 20/21, 95.2%, respectively). The ileocecal and colonic regions are common sites of CD with a relatively fixed anatomical position, and usually, the lesions are more severe than in other regions, which may lead to higher interobserver consistency.

CF plays an active role in the pathogenesis of CD [1, 15–17]. In previous studies, the CF represented by mesenteric creeping fat index (MCFI) was significantly positively correlated with collagen fiber deposition and smooth muscle hyperplasia/hypertrophy on surgical specimens (*r* = 0.568, *p* = 0.000) [3]. On this basis, our study further included non-surgical CD patients with different disease behaviors, which was in line with the actual clinical situation and greatly expanded the applicability of non-invasive imaging evaluation of CF. Although the pathological and physiological manifestations were not completely the same, CF showed in both intestinal fistula and intestinal stenosis. However, it could not be accurately distinguished on imaging. Unlike previous studies, we included patients with concurrent intestinal fistulas, and the value of CF in stenosis and intestinal fistulas needs further investigation. The high agreement between IUS and CTE observed for the thickest bowel segment suggested that IUS could be useful to objectively measure CF in clinical studies, along with CTE. Therefore, IUS could be a helpful tool for assessment and monitoring during the treatment of CD.

Our study had some limitations. First, this was a retrospective study conducted at a single academic institution. Selection bias might have occurred mainly because a large proportion of patients with moderate and severe disease were included. Second, the number of enrolled patients was small. Therefore, larger, multicenter studies are needed to confirm our conclusions.

## Conclusion

This study helped to standardize the use of CF for reporting IUS in CD patients. IUS could be of value and complementary to CTE in assessing CF, particularly in patients with lesions in the terminal ileal and colon, and as a non-invasive technique for monitoring CD patients.

## Data Availability

The data generated during the current study are available from the corresponding author upon reasonable request.

## Abbreviations

CTE: Computed tomography enterography
CF: Creeping fat
CD: Crohn’s disease
MCFI: Mesenteric creeping fat index
US: Ultrasound
